# Securing IoT Networks Against DDoS Attacks: A Hybrid Deep Learning Approach

**DOI:** 10.3390/s25051346

**Published:** 2025-02-22

**Authors:** Noor Ul Ain, Muhammad Sardaraz, Muhammad Tahir, Mohamed W. Abo Elsoud, Abdullah Alourani

**Affiliations:** 1 Department of Computer Science, COMSATS University Islamabad, Attock Campus, Attock 43600, Pakistan; sp23-rcs-005@cuiatk.edu.pk (N.U.A.); m_tahir@cuiatk.edu.pk (M.T.); 2 Department of Computer Science and Information, College of Science at Zulfi, Majmaah University, P.O. Box 66, Al-Majmaah 11952, Saudi Arabia; m.wagieh@mu.edu.sa; 3 Faculty of Computers and Informatics, Suez Canal University, Ismailia 41522, Egypt; 4 Department of Management Information Systems, College of Business and Economics, Qassim University, Buraydah 51452, Saudi Arabia; ab.alourani@qu.edu.sa

**Keywords:** deep learning, convolutional neural networks, classification accuracy, Internet of Things, DDoS

## Abstract

The Internet of Things (IoT) has revolutionized many domains. Due to the growing interconnectivity of IoT networks, several security challenges persist that need to be addressed. This research presents the application of deep learning techniques for Distributed Denial-of-Service (DDoS) attack detection in IoT networks. This study assesses the performance of various deep learning models, including Latent Autoencoders, LSTM Autoencoders, and convolutional neural networks (CNNs), for DDoS attack detection in IoT environments. Furthermore, a novel hybrid model is proposed, integrating CNNs for feature extraction, Long Short-Term Memory (LSTM) networks for temporal pattern recognition, and Autoencoders for dimensionality reduction. Experimental results on the CICIOT2023 dataset show the enhanced performance of the proposed hybrid model, achieving training and testing accuracy of 96.78% integrated with 96.60% validation accuracy. This presents its efficiency in addressing complex attack patterns within IoT networks. Results’ analysis shows that the proposed hybrid model outperforms the others. However, this research has limitations in detecting rare attack types and emphasizes the importance of addressing data imbalance challenges for further enhancement of DDoS attack detection capabilities in future.

## 1. Introduction

The Internet of Things (IoT) has brought a revolution in many domains, such as smart homes, industrial automation, and healthcare. However, this interconnectedness also introduces significant security challenges [1]. The increase in IoT has led to a significant increase in the number and sophistication of IoT attacks, posing threats to privacy, data integrity, and overall cybersecurity [2]. The significance of IoT security lies in various aspects of daily life. IoT devices are integrated into smart homes, cities, industrial systems, and healthcare, making their security crucial to protect against widespread implications and ensure safe operation [3]. These devices often handle sensitive personal data, necessitating robust security measures to protect privacy and maintain data integrity, which is essential for ensuring user trust in IoT technologies [4]. The interconnected nature of IoT devices creates a broad range of attacks for attackers. Effective detection and mitigation strategies are necessary to counter the increasing IoT attacks, such as DDoS, as well as protection against potential disruptions and damages [5]. Ensuring robust IoT security is vital to protect public safety and security from potential catastrophic consequences of successful attacks [6]. Additionally, the continuous evolution of the IoT landscape introduces new vulnerabilities, making ongoing research and development in IoT security essential to keep up with technological advancements and protect against emerging threats.

Existing solutions for IoT security include edge and fog computing, which shift data processing and analytics closer to data sources, reducing latency and enabling real-time threat detection and responses [7]. This approach also offloads the computational burden from IoT devices to more capable edge or fog nodes. Blockchain technology enhances IoT security by providing a decentralized and tamper-proof ledger for logging device interactions, ensuring data integrity and authenticity, and making it difficult for attackers to manipulate data [8]. Lightweight cryptography, designed specifically for resource-constrained IoT devices, provides essential security functions such as encryption, authentication, and data integrity without imposing significant computational overhead [9]. Anomaly detection systems monitor IoT network traffic for unusual patterns that may indicate security breaches, and machine learning-based anomaly detection models can identify and alert of potential threats in real time, even for unknown attacks [10].

Despite significant advancements in IoT security, several areas remain that need further exploration. Scalability and generalization are major concerns, as many security solutions show promise in controlled environments or specific datasets, but their effectiveness in diverse and large-scale real-world IoT deployments remains uncertain [11]. Interoperability and standardization pose challenges due to the lack of uniformity across IoT devices and communication protocols, creating significant security gaps [12]. Ensuring interoperability while maintaining robust security requires the development of universal standards that can be widely adopted without compromising security. Resource constraints are another issue, as many IoT devices have limited computational power, memory, and energy resources, making it difficult to implement sophisticated security mechanisms [13]. Research should focus on developing lightweight yet effective security algorithms that can operate within these constraints. Innovative approaches are needed to ensure timely mitigation of threats without overwhelming resources [14]. Lastly, adaptive and evolving threats need continuous research, as cyber threats are continually evolving, with attackers developing new methods to bypass existing security measures. The ability of security solutions to adapt to new and sophisticated attacks is a critical area that requires further investigation [15].

This article presents a hybrid deep learning model for DDoS attack detection in IoT networks using the CICIOT2023 dataset [16]. The model involves preprocessing the dataset, selecting relevant features, and training machine learning models. These models are evaluated and fine-tuned for DDoS detection, using patterns and anomalies in network traffic. The approach utilizes deep neural networks to learn patterns and features from the preprocessed dataset. Finally, a hybrid approach is used by integrating Autoencoder, LSTM and a CNN. Each component addresses specific challenges in IoT attack detection, using the insights provided by the CICIOT2023 dataset. Autoencoders learn the structure of normal traffic and detect anomalies via reconstruction errors, LSTM captures temporal patterns and dependencies, and a CNN identifies spatial relationships, such as packet sizes and protocol flags. Together, these models enable the detection of complex attack patterns in network traffic. The proposed model is evaluated using accuracy, precession, recall, and F-1 score. The results are compared with state-of-the-art models. This research investigates how the integration of Autoencoders, LSTM, and a CNN in a hybrid model can improve the accuracy and efficiency of DDoS attack detection, explore how the hybrid model adapts to evolving attack patterns and variations in IoT network traffic.

The rest of this paper is organized as follows: Section 2 presents related work, including machine learning, deep learning, and hybrid approaches. Section 3 presents the proposed model, including details of different phases, followed by results and discussion in Section 4. Section 5 concludes this article.

## 2. Related Work

There many studies in the literature that support intrusion detection in IoT networks. Many researchers generate and utilize different datasets for attack detection and mitigation. The three main categories include machine learning-based methods, deep learning-based methods, and hybrid methods. This section presents a comprehensive review and comparative analysis of different models in these categories.

### 2.1. Machine Learning Methods

Nguyen et al. presented a survey and identified weaknesses in federated learning-based intrusion detection, particularly backdoor attacks [17]. Their work emphasizes the necessity of robust defenses in federated learning environments to ensure IoT security, highlighting the importance of advanced security measures to mitigate potential risks. A multi-layer framework integrating network traffic analysis across diverse network entities is proposed in [18]. This approach yields better accuracy in detecting DDoS attacks within IoT environments, highlighting the effectiveness of multi-layered detection strategies. Another study introduced a multi-view federated method using ensemble learning and artificial neural networks (ANNs) trained on diverse traffic features [19]. The authors employed Grey Wolf Optimization (GWO) for feature selection, which reduced memory requirements while maintaining accuracy. However, their evaluation lacked IoT-specific DDoS attack data, indicating a need for further refinement and validation in real-world scenarios. Another system addresses the challenge of detecting DDoS attacks in IoT networks using a hybrid methodology [20]. The methodology combines feature selection methods (chi-square, Extra Tree, ANOVA) with machine learning classifiers, i.e., Random Forest (RF), Decision Tree (DT), K-Nearest Neighbors (KNN), XGBoost). The CICDDoS2019 dataset trains and evaluates the system in a cloud-based environment. Syed et al. [21] proposed a machine learning-based framework for detecting application layer DoS attacks on the MQTT protocol. The experiments show that the framework effectively identifies attacks and reduces false positives by utilizing features like field size and length. The results demonstrate that attackers can overwhelm MQTT brokers, even under resource restrictions. The proposed system maintains high detection accuracy. Authors in [22] focused on detecting DDoS attacks in banking sector using the Banking Dataset. They evaluate multiple classification models, including support vector machine (SVM), KNN, and RF. Authors in [23] tackle the persistent challenge of DDoS attacks on IoT devices by proposing a hybrid methodology that utilizes feature selection techniques and machine learning classifiers for detection. Authors in [24] present a machine learning pipeline designed to detect DDoS attacks in IoT networks, addressing the increased vulnerability of these interconnected systems. The proposed approach includes a data processing module, a dynamic attribute selection module to enhance feature selection and reduce training time, and a classification module for attack detection. Using the CICI-IDS-2018 dataset, the authors evaluated five classifiers, i.e., DT, Gaussian Naive Bayes, LR, KNN, and RF.

### 2.2. Deep Learning Methods

This section presents DDoS attack detection based on deep learning. Authors in [25] proposed a model to detect DDoS attacks in IoT devices. The paper proposes a DDoS detection system using deep neural networks (DNN) and LSTM algorithms, applied to an improved CICDDoS2019 dataset with a new taxonomy for DDoS attacks. ElSayed et al. [26] introduced DDoSNet, a deep learning-based intrusion detection system designed to address DDoS attacks in Software-Defined Networking (SDN) environments. By separating the control and data planes, the proposed DDoSNet model combines recurrent neural networks (RNNs) with Autoencoders and is evaluated using the CICDDoS2019 dataset, which includes a broad range of DDoS attack patterns. The results show significant improvements in attack detection over existing methods, demonstrating the effectiveness of deep learning in securing SDN networks. Authors in [27] proposed anomaly-based intrusion detection models utilizing CNN along with transfer learning techniques. The method showed better results, particularly in multi-class and binary classification for IoT intrusion detection. In another study [28], the authors introduced a federated transfer learning approach for intrusion and attack detection in mobile computing environments. Combining transfer learning with CNN achieved better performance and reduced computational costs. Challenges remain in addressing the dynamic nature of IoT environments and online attack detection. Zeeshan et al. [29] proposed a Protocol-Based Deep Intrusion Detection (PB-DID) architecture for DoS and DDoS attacks in IoT networks. The approach involves creating a dataset from IoT traffic by integrating features from the UNSW-NB15 and Bot-IoT datasets, focusing on flow and TCP characteristics. The architecture classifies traffic into non-anomalous, DoS, and DDoS categories while addressing issues like class imbalance and overfitting. Authors in [30] address the issue of DoS and DDoS attacks on IoT devices. Utilizing the Bot-IoT dataset, the researchers tackled the class imbalance problem and proposed a detection model using machine learning and deep learning. They experimented with three different feature sets for binary and multiclass classifications, achieving better accuracy. Comprehensive evaluations revealed that the DT and Multi-layer Perceptron models were the most effective in identifying DDoS and DoS attacks. Authors in [31] explore the detection of DDoS attacks. The study employs four machine learning classifiers KNN, DT, RF, and ANN to classify normal and DDoS traffic. Using the CICDDoS2019 dataset, the researchers found that ANN outperformed the other models, delivering the best results in detecting DDoS attacks. Maseer et al. [32] conduct a comprehensive review of anomaly-based intrusion detection systems (AIDS) using machine and deep learning algorithms. They identify issues in previous studies, such as randomness in selecting algorithms, outdated datasets, and shallow analyses. To address these, the study evaluates 10 popular supervised and unsupervised machine learning algorithms, including ANN, DT, KNN, Naive Bayes (NB), RF, SVM, CNN, expectation-maximization (EM), k-means, and self-organizing maps (SOMs).

### 2.3. Hybrid Methods

This section presents details of the hybrid methods used for DDoS attack detection in IoT networks. Zainudin et al. [33] present a hybrid CNN-LSTM model with XGBoost feature selection for DDoS attack classification, achieving better accuracy. The proposed model highlights the potential effectiveness of hybrid models in mitigating DDoS attacks in IoT networks. Authors in [34] address the limitations of supervised learning models in detecting unknown DoS/DDoS attacks in IoT by proposing a hybrid detection approach. The method combines a soft-ordering CNN (SO-CNN) with local outlier factor (LOF) and isolation-based anomaly detection using nearest-neighbor ensembles (NNEs). Evaluated on BoT-IoT, CIC-IDS-2017, and CIC-IDS-2018 datasets, the proposed model achieves better accuracy. In another study [35], the authors present a novel deep learning-based intrusion detection system for cloud or fog deployment in IoT environments. The hybrid model integrates a CNN, LSTM, Deep Autoencoder, and DNN into a two-level architecture. Evaluated using the CIC-DDoS2019 dataset, the model demonstrated better performance compared to traditional machine learning and deep learning methods, achieving high true-positive rates, accuracy, and low false alarm rates. Authors in [36] proposed a hybrid intrusion detection architecture for the fog computing layer. The approach, called DNN-KNN, combines DNN with KNN for binary classification of attacks or normal. The proposed model is evaluated with NSL-KDD and CICIDS2017 datasets. The hybrid approach not only offers high accuracy and recall rates but also operates with low memory and processing overhead, demonstrating significant improvements over traditional intrusion detection methods. To address the challenges of attack detection, Aswad et al. [37] proposed a deep learning-based DDoS detection model that combines RNN, LSTM, and a CNN into a bidirectional CNN-BiLSTM architecture. The model is trained and tested using the CICIDS2017 dataset. Authors in [38] proposed an intrusion detection system for SDN utilizing a Gated Recurrent Unit Recurrent Neural Network (GRU-RNN). Better results are achieved in terms of accuracy. Hossain et al. [39] explore an LSTM-based approach for network attack detection. By fine-tuning various LSTM hyperparameters, optimizers, loss functions, learning rates, and activation functions, the study aims to identify optimal configurations for detecting network attacks using the CICIDS 2017 labeled dataset. Authors in [40] employ LSTM and RNN within the TensorFlow framework to develop an intrusion detection system for detecting DDoS attacks. Authors in [41] present a deep learning-powered intrusion detection system specifically designed for detecting and mitigating DDoS attacks in IoT networks. Using the CICIoT2023 dataset, evaluates different deep learning models, including DNN, CNN, and LSTM to enhance intrusion detection performance. Features extraction, duplication removal, normalization, and random subset selection improve the quality of the dataset. Balanced datasets are created to reduce class imbalance, leading to better model generalization. Nkoro et al. [42] proposed a zero-trust framework for marine cybersecurity, utilizing Explainable Artificial Intelligence (XAI) to address the lack of transparency and trustworthiness in traditional intrusion detection systems. The framework aims to detect and mitigate cyberattacks in maritime environments with high accuracy and interpretability. The framework is evaluated using the 2023 EdgeIIoTset and CICIoT2023 datasets, which include diverse IoT-based attack scenarios. Authors in [43] introduced a lightweight deep learning-based intrusion detection system called DL-BiLSTM, which integrates DNN and Bi-LSTM for enhanced feature extraction. The model further incorporates Incremental Principal Component Analysis (IPCA) for feature reduction and dynamic quantization to minimize computational complexity, making it suitable for real-time IoT security. The proposed model uses CICIDS2017, N-BaIoT, and CICIoT2023 datasets for training and evaluation. Data preprocessing includes numerical encoding, normalization, and feature reduction. Abbas et al. [44] explore the use of deep learning models, including DNN, CNN, and RNN, for cyberattack detection in IoT networks. The CICIoT2023 dataset is used for evaluation of the models.

In the context of IoT security, detecting DDoS attacks requires advanced intrusion detection systems (IDSs) due to the inadequacy of traditional signature-based and anomaly-based methods against evolving threats. Machine learning-based methods have achieved better results by incorporating optimization techniques [17,18,19]. Hybrid methodologies have also shown better results. Deep learning methods have demonstrated better detection capabilities for DDoS attacks, utilizing DNN, LSTM, and CNN approaches [23,24,25]. Researchers also explored federated transfer learning and protocol-based deep intrusion detection for improved IoT security [26,27]. Models such as CNNs and LSTM networks have demonstrated better performance in analyzing both spatial and temporal features of network traffic, making them particularly effective to identify sophisticated attack patterns. Hybrid deep learning architectures, such as CNN-LSTM and bidirectional CNN-BiLSTM, further enhance detection capabilities by combining the strengths of different model types, achieving better accuracy and low false alarm rates. Hybrid learning approaches have gained significant attention in the field of DDoS attack detection for IoT networks due to the ability to combine the strengths of multiple methodologies, resulting in improved detection accuracy and robustness. These models integrate different machine learning and deep learning techniques to utilize advantages such as the efficiency of machine learning algorithms in feature selection and the advanced pattern recognition capabilities of deep learning models. By integrating anomaly detection techniques, such as Isolation Forests or Local Outlier Factors (LOF), hybrid approaches enhance the ability to detect unknown or zero-day attacks that cannot be identified using traditional signature-based systems. In the context of IoT, hybrid models have demonstrated significant success in adapting to the dynamic nature of network traffic by combining supervised and unsupervised learning methods.

Existing methods for detecting and mitigating DDoS attacks often rely on traditional techniques, such as statistical analysis, signature-based approaches, or simple machine learning models. Although these methods can detect certain types of attacks, they struggle to accurately capture the complex, bidirectional nature of network traffic, especially in the case of sophisticated, evolving attacks. Conventional approaches typically fail to extract the deep, temporal, and spatial features that are important to identify subtle and dynamic attack patterns. Many existing models are unable to effectively capture the time-varying characteristics of network traffic, making it challenging to detect attacks that evolve over time or are disguised within legitimate traffic. The gap in feature extraction and the inadequacy of existing methods have led to suboptimal detection rates. As a result, there is a need for advanced methods that can overcome these limitations, particularly through the use of deep learning techniques that integrate both temporal and spatial features, and pattern recognition, enabling a more comprehensive and accurate understanding of network traffic. There is a need to focus on extracting both the low-level and high-level features from network traffic and combine them in hybrid models to enhance the accuracy of DDoS detection attacks.

## 3. Materials and Methods

This research utilizes a hybrid deep learning model combining Autoencoders, LSTM networks, and CNNs to detect DDoS attacks in IoT networks. Autoencoders learn the structure of normal traffic and detect anomalies via reconstruction errors, LSTM captures temporal patterns and dependencies, and a CNN identifies spatial relationships, such as packet sizes and protocol flags. Together, these models enable the detection of complex attack patterns in network traffic.

The hybrid architecture concatenates the features extracted by each model into a unified feature vector. The Autoencoder highlights normal traffic patterns, the LSTM processes 3D reshaped data through sequential layers to extract temporal features, and the CNN applies convolutional filters and max-pooling to capture local dependencies. The combined features are passed through fully connected layers with dropout for refinement and classification into normal or attack traffic.

The model is trained using the Adam optimizer and categorical cross-entropy loss, with hyperparameters fine-tuned for optimal performance. Evaluation metrics, i.e., accuracy, precision, recall and the F1-score, are used to assess the effectiveness on a test dataset. Using spatial and temporal features, the hybrid model achieves robust and accurate detection of DDoS attacks while minimizing false positives and negatives, making it highly effective in IoT environments. The integration of multiple feature extraction techniques ensures the robustness of the model and adaptability, making it well suited for dynamic and resource-constrained nature of IoT environments.

**Figure 1:**
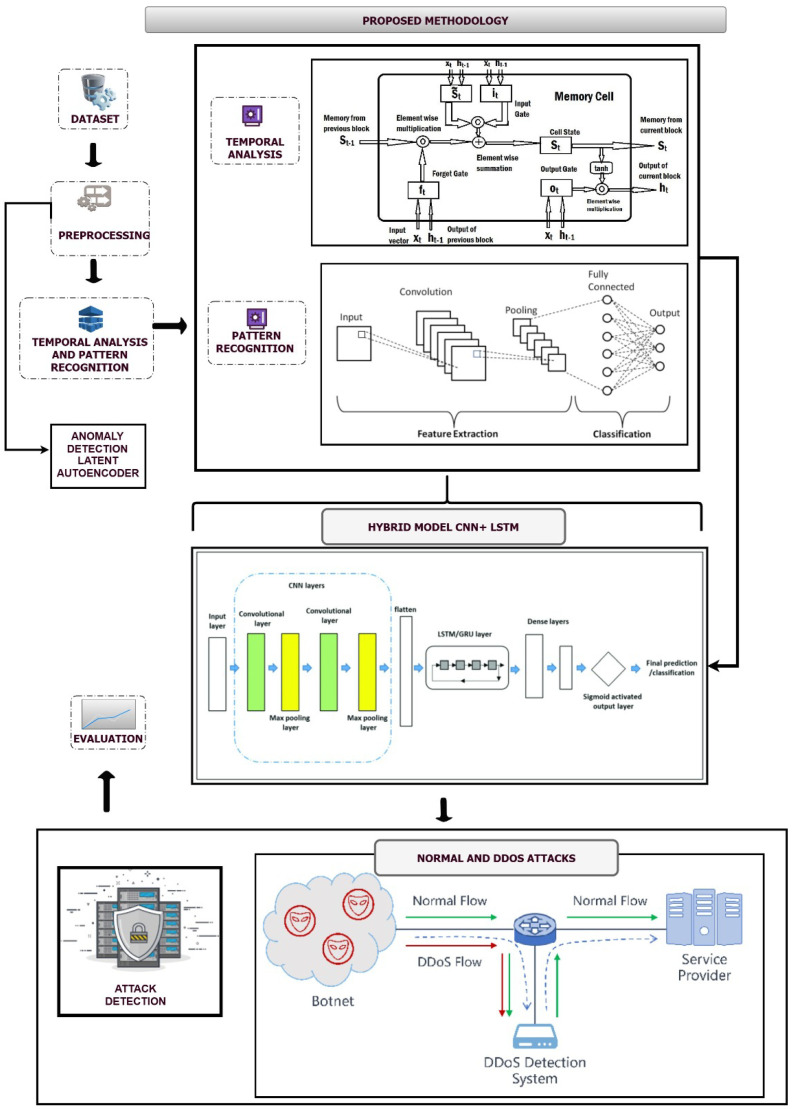
Workflow of the proposed model.

This research uses a quantitative, experimental design to develop and evaluate a hybrid model for detecting DDoS attacks in IoT networks. The methodology integrates traditional machine learning techniques with deep learning models, specifically Autoencoders, LSTM networks, and CNNs, to analyze network traffic patterns and detect anomalies. The methodology consists of multiple stages, including data preprocessing, feature extraction, and model integration, which are outlined in detail, as shown in Figure 1.

### 3.1. Dataset

The CICIoT2023 dataset includes labeled samples, where each sample represents a network observation with a set of features and a classification label. The labels indicate whether the sample belongs to the “Normal” class (benign activity) or the “Attack” class (anomalous or malicious activity). The dataset captures 33 distinct attack scenarios across seven categories i.e., DDoS, DoS, reconnaissance, brute force, spoofing, web-based attacks, and Mirai botnet attacks. The dataset is organized in a tabular format, where rows represent individual samples and columns denote specific features. These features, derived from network traffic and other sources, include sensor readings, statistical measures, and derived metrics, enabling the classification of normal and attack patterns.

### 3.2. Data Preprocessing

Data preprocessing plays a crucial role in preparing the dataset for model training. Raw data may contain inconsistencies, missing values, or noise that could potentially reduce the performance of the models. Therefore, the dataset undergoes several preprocessing steps—including the standardization of features to ensure that they are on the same scale—and encoding of categorical variables. The data are also split into training and testing sets to allow for proper model evaluation. This division ensures that the model can be trained on one portion of the dataset and then evaluated on an unseen portion, thereby helping to assess its generalization capability. To handle the high-dimensional nature of IoT data, dimensionality reduction techniques and feature engineering were applied to focus on the most relevant attributes for classification.

- Data Standardization
  Standardization is particularly important when the features vary in scale or range, as neural networks and other machine learning algorithms are sensitive to differences in feature magnitudes. Features with larger ranges can dominate the learning process, causing slower convergence or leading the model to focus disproportionately on certain features. To prevent this, all features are standardized. This is achieved using the StandardScaler method.
- Categorical Data Encoding In the dataset, the target variable is categorical, consisting of two classes, i.e., normal and attacks. Machine learning models, especially those used for deep learning tasks, require numerical data as input, meaning that categorical variables must be encoded appropriately before they can be processed. To achieve this, label encoding and one-hot encoding techniques are employed. Label encoding converts each class label into a unique integer, which is suitable for models that can handle numerical labels directly. The class labels are further one-hot encoded, transforming the categorical labels into a binary matrix where each class is represented by a vector of 0 s and 1 s.
- Data Splitting
  Once the data are cleaned and encoded, the next step is to split the dataset into training and testing sets. The training set is used to train the machine learning models, while the testing set serves as a holdout set to evaluate model performance on unseen data. The dataset is typically divided into 80% for training and 20% for testing, although other ratios can be used depending on the size of the dataset. A stratified sampling approach is used to ensure that both the “normal” and “attack” classes are proportionally represented in both the training and testing sets. This ensures that the model is trained on a balanced dataset and can generalize better when making predictions on new or unseen data.
- Feature Selection and Dimensionality Reduction
  To enhance the efficiency of the model and prevent overfitting, feature selection techniques are applied. Given the high dimensionality of the dataset, some features may be redundant or irrelevant to the classification task. Therefore, feature selection is performed to retain only the most significant and informative features. Additionally, dimensionality reduction techniques, such as Principal Component Analysis (PCA), are employed to reduce the number of features while preserving the variance in the data. The dataset contains 46 features. Features with zero or null values were removed, and 37 features were included. Through these data-cleaning and preparation steps, the dataset was transformed into a suitable format for training machine learning models. Each step ensures that the data are consistent, standardized, and appropriately encoded, thereby enabling the models to learn from high-quality input. As a result, the research methodology becomes more robust and reliable, with a strong foundation of well-prepared data that can lead to accurate and meaningful results.

### 3.3. Features Extraction

Feature extraction plays a critical role in enhancing the predictive power of machine learning models, especially when dealing with high-dimensional data. In the context of this research, feature extraction involves using various deep learning techniques to transform the raw input data into more informative representations that can be effectively used for classification tasks. The goal is to extract relevant features from the dataset that capture essential patterns while reducing the complexity of the data. This process also aims to minimize the risk of overfitting by focusing on the most significant aspects of the data. The following sections outline the methods used for feature extraction in this study, which includes Autoencoders, LSTM networks, and CNNs as individual feature extractors. These techniques are combined into a hybrid model to use the strengths of each method.

#### Feature Extraction

This subsection presents feature extraction with different models, including the Autoencoder, CNN, LSTM and hybrid model.

- Autoencoder
  The architecture of the Autoencoder in this study consists of multiple layers, starting with a dense input layer followed by two hidden layers with ReLU activation functions, aimed at progressively compressing the data. The latent space, or the bottleneck layer, is set to a size of 10 to capture the most significant features. The decoder part of the Autoencoder then reconstructs the original data from the latent space, ensuring that the encoder extracts the features necessary for accurate data representation. The Autoencoder is trained using mean squared error (MSE) as the loss function, optimizing it to produce a compact yet meaningful feature representation. The encoder is used as a feature extractor, which outputs the learned latent features that can be passed to subsequent models.
- LSTM
  In this study, the LSTM feature extractor is designed to process the input data reshaped into 3D arrays, where each sample is treated as a sequence of features. The model consists of two LSTM layers: the first with 32 units, followed by a second LSTM layer with 16 units. Both layers use ReLU activation functions and are followed by dropout layers to prevent overfitting. The output of the final LSTM layer is a compressed representation of the input data, which is then used as a feature vector for further classification. The LSTM-based feature extraction allows the model to capture any hidden patterns or interactions between features, potentially improving the ability of the model to classify complex data.
- CNN
  The CNN feature extractor is designed to operate on the reshaped input data, where each sample is treated as a sequence of features in a one-dimensional structure. The CNN model consists of two convolutional layers followed by max-pooling operations. The first convolutional layer uses 32 filters with a kernel size of 1, followed by a second convolutional layer with 64 filters. The output of the convolutional layers is then passed through a max-pooling operation with a pool size of 1, effectively reducing the dimensionality while retaining key features. Finally, the output is flattened and passed to the next layers of the network. By using a CNN for feature extraction, the model can identify hierarchical relationships within the data, allowing it to learn more discriminative features that are useful for classification tasks.
- Hybrid Model
  To maximize the benefits of the individual feature extractors described above, a hybrid model is employed, which combines the extracted features from the Autoencoder, LSTM, and CNN. Each of these models extracts complementary features from the data. By concatenating the outputs, the hybrid model can utilize the strengths of each individual model. This approach allows the model to learn both global and local patterns in the data, as well as long-range dependencies, resulting in a more robust and comprehensive feature representation. The working of the hybrid model starts by taking the output features from each of the individual models. These features are then concatenated into a single feature vector, which serves as the input to a fully connected neural network for classification. The concatenation of features ensures that the model can benefit from the unique capabilities of each individual model, combining them into a powerful feature representation. The fully connected layers that follow the concatenation are designed to further refine these features, with dropout layers applied to prevent overfitting and improve generalization.

### 3.4. Anomaly Detection

In this study, the Autoencoder is also utilized for anomaly detection, particularly focusing on identifying potential DDoS attacks within network traffic. Autoencoders, being unsupervised models, are capable of learning the normal patterns of the data without requiring labeled attack data. Specifically, the Autoencoder is trained exclusively on non-attack (normal) network traffic, where it learns the typical behavior patterns present in the system. Once the Autoencoder is trained, it can be applied to new incoming network traffic data. The model then attempts to reconstruct the data, comparing the original input with its reconstruction. If the data point does not match the expected pattern, i.e., there is a significant deviation in the reconstruction error, this is flagged as an anomaly. DDoS attacks, being distinct and abnormal patterns in the traffic, typically exhibit high reconstruction errors because they deviate from the learned normal behavior. Therefore, anomalies are detected when the reconstruction error exceeds a predefined threshold, signaling potential attack traffic.

Following the training of the Autoencoder on normal network traffic data, anomaly detection is performed by calculating the reconstruction error for each data sample. The reconstruction error is computed as the difference between the input data and the reconstructed output. This error indicates how well the Autoencoder has learned to represent the normal traffic patterns. For a given sample, if the reconstruction error is relatively low, it implies that the sample closely matches the normal traffic patterns. Thus, it is considered a normal instance. On the other hand, a higher reconstruction error shows that the sample deviates significantly from the learned patterns and is flagged as an anomaly. The reconstruction error serves as a robust indicator of the presence of potential DDoS attacks or any other irregular traffic behavior. To effectively detect anomalies, a threshold is established based on the distribution of reconstruction errors in the training data. One common approach is to use the 95th percentile of the reconstruction error distribution, setting the threshold so that only the top 5% of errors are classified as anomalies. Any sample with a reconstruction error greater than this threshold is identified as a potential anomaly, which could be indicative of a DDoS attack.

### 3.5. Temporal Analysis

To capture these temporal dependencies, LSTM networks, a specialized type of recurrent neural network (RNN), are utilized. LSTMs are designed to model sequential data by capturing long-term dependencies and remembering past information for longer periods, which is particularly useful for time-series data like network traffic.

In this study, the network traffic data are segmented into time windows, where each window represents a sequence of traffic observations over a specific time period. These sequences are then fed into the LSTM model. The LSTM is trained to learn the temporal patterns in the data such as sudden spikes in traffic volume or repetitive requests that indicate an ongoing attack. By learning these time-based patterns, the LSTM model can effectively identify anomalies based on unexpected changes in network behavior, such as a sudden increase in traffic or the repetition of specific patterns indicative of a DDoS attack. The LSTM model enhances anomaly detection by accounting for the temporal relationships in the data, which can be crucial for detecting time-sensitive attacks that may not be easily identified using static feature extraction methods.

### 3.6. Pattern Recognition

In this study, a 1D CNN is employed to detect spatial patterns within network traffic data, focusing on learning from features in a sequential format without the need to convert the data into 2D representation. The CNN approach is particularly effective for identifying patterns in data sequences, and, in this context, it is used to detect anomalies that may indicate DDoS attacks. The primary advantage of using a 1D CNN lies in its ability to capture spatial relationships within sequential data. Unlike traditional CNNs that are typically used for 2D image data, a 1D CNN applies convolutional filters along a single dimension, allowing it to process time-series data or feature sequences efficiently. In the context of network traffic, such data often include sequential information, such as packet sizes, inter-arrival times, or protocol flags, which are crucial for identifying irregular patterns.

The 1D CNN model is applied directly to the network traffic features that have been preprocessed and organized into sequential data. The model architecture consists of multiple convolutional layers that progressively learn increasingly complex features within the network traffic. Each convolutional layer applies filters across the feature dimension to capture spatial patterns, such as periodic increases in traffic volume or repetitive packets associated with attack behaviors. The 1D CNN can, therefore, detect low-level features like fluctuations in packet size, as well as higher-level patterns that signify attack behaviors. To enhance the ability of the model to generalize and recognize patterns effectively, the network also includes pooling layers that help reduce the dimensionality and computational load. These pooling layers serve to extract the most significant features from each convolutional operation, preserving the key information while minimizing the risk of overfitting. After the convolutional and pooling layers, the output is flattened and passed through fully connected dense layers, where it is classified as either normal or anomalous (indicating a potential DDoS attack).

### 3.7. Hybrid Model Integration

The integration of the Autoencoder, LSTM, and 1D CNN models forms a hybrid system that comprehensively analyzes network traffic data to detect anomalies, including potential DDoS attacks. Each individual model contributes a unique aspect of feature extraction whether it be capturing deviations from normal behavior, temporal dependencies, or spatial patterns resulting in a powerful system capable of more accurate anomaly detection.

In the hybrid model, features extracted by the Autoencoder, LSTM, and 1D CNN are concatenated into a single unified feature vector. The Autoencoder is responsible for detecting anomalies by learning the typical patterns of normal network behavior and identifying any deviations during reconstruction. The LSTM captures temporal dependencies and time-based patterns in the sequential data, allowing the model to identify unusual trends or fluctuations over time that may indicate an ongoing attack. Finally, the 1D CNN focuses on spatial patterns, detecting local relationships between features in the network traffic that are indicative of specific attack behaviors, such as the structure of packet sequences or fluctuations in protocol flags.

Once these diverse feature sets are combined, they are passed through a final fully connected dense layer, which is trained to differentiate between normal and attack traffic. This final layer performs the classification task, using the integrated feature set to classify the data as either “normal” or “attack”. The advantage of combining these feature sets is that the hybrid model can learn from different perspectives of the data, capturing a broader range of characteristics of network traffic.

By integrating the strengths of these three models, the hybrid system provides a robust approach to anomaly detection. The model can detect a wide variety of DDoS attack types that might exhibit different characteristics in terms of timing, spatial patterns, or behavior over time. Moreover, the hybrid approach enhances the ability of the model to generalize across various attack scenarios, reducing the likelihood of false positives and ensuring more accurate identification of abnormal network behavior.

### 3.8. Model Training

Training the hybrid model involves several key steps that ensure the model effectively learns to distinguish between normal and attack traffic patterns. The Adam optimizer, known for its efficient performance in deep learning tasks, is utilized to update the weights of the model during training. Adam adapts the learning rate based on the training process, making it particularly useful for complex, non-linear optimization tasks, such as training neural networks. The categorical cross-entropy loss function is chosen for this multi-class classification problem since the labels are one-hot encoded. This loss function measures the difference between the predicted and true class distributions and is widely used in classification tasks where the target variable consists of multiple classes.

The model is trained for several epochs to ensure it learns the underlying patterns in the data. During training, the model is exposed to both normal and attack traffic samples, allowing it to learn the differences between the two. To avoid overfitting, which can occur when the model learns to perform well only on the training data but fails to generalize to new data, early stopping is employed. This technique monitors the performance of the model on a validation set during training and halts training when the performance of the model stops improving, ensuring it does not memorize the training data.

### 3.9. Model Evaluation

After the training phase, it is necessary to evaluate the performance of the model on an independent test dataset. This evaluation provides a clear indication of how well the model can generalize to new data and its effectiveness in distinguishing between normal and attack traffic. The evaluation process involves the use of several performance metrics, including accuracy, precision, recall, and F1-score, which provide a comprehensive view of the capabilities of the model.

## 4. Results and Discussion

This section presents the performance evaluation of the proposed hybrid models for detecting DDoS attacks. The models examined include the Latent Autoencoder, LSTM, CNN, and Hybrid architecture that combines CNN, LSTM, and Autoencoder layers. Through extensive experimentation, the models were assessed in terms of accuracy, precision, recall, F1-score, and loss, providing insights into their capabilities for distinguishing between benign traffic and various DDoS attack types.

Accuracy: Accuracy shows the overall effectiveness of the classification model. It considers both correctly classified true-positive (TP) and true-negative (TN) instances, divided by the total number of instances (including false positives and false negatives). Equation (1) is used to calculate the accuracy.

(1) $$Accuracy=\frac{TP+TN}{TP+TN+FP+FN}$$

Precision: Precision measures the proportion of actual true-positive cases (TP) among the instances classified as positive by the model. It helps identify how well the model avoids false positives. Precision is calculated using Equation (2), as follows:

(2) $$Precision=\frac{TP}{TP+FP}$$

Recall: Recall represents the ability of the model to identify all relevant true-positive cases (TP). It reflects how well the model avoids false negatives. Equation (3) is used to calculate the recall.

(3) $$Recall=\frac{TP}{TP+FN}$$

F1-Score: The F1-score is a harmonic mean between precision and recall, providing a balanced view of both metrics. It is useful when a single metric is desired to capture both precision and completeness. The F1-score is calculated using Equation (4), as follows:

(4) $$F_{1}Score=\frac{2\cdot \mathrm{Precision}\cdot \mathrm{Recall}}{\mathrm{Precision}+\mathrm{Recall}}$$

### 4.1. Latent Autoencoder

**Figure 2:**
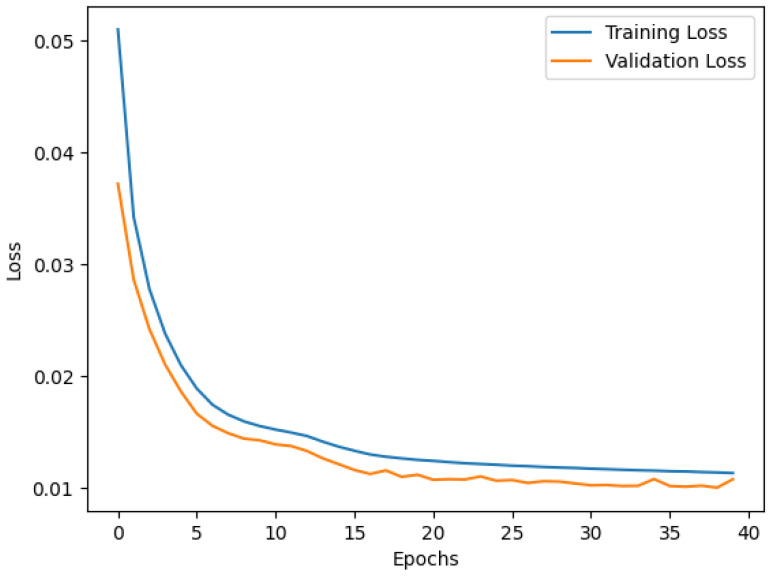
Validation and training loss of Autoencoder.

The Autoencoder was trained and evaluated to detect anomalies by learning to reconstruct the patterns of normal data. The training and validation loss curves in Figure 2 show the learning process of the model over 40 epochs. Initially, the losses decreased rapidly, reflecting the ability of the model to learn key data representations effectively. By the final epoch, the training loss reached 0.10 and the validation loss reached 0.08, demonstrating strong generalization and low overfitting.

**Figure 3:**
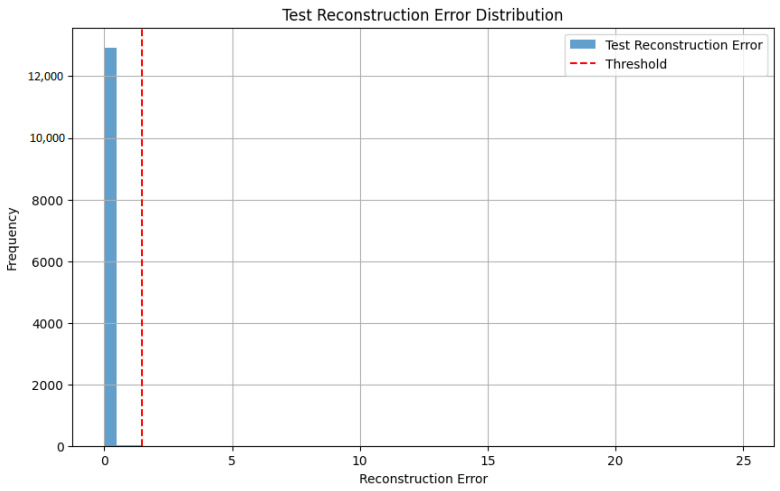
Distribution of reconstruction errors.

To detect anomalies, a threshold for reconstruction error was established at 1.4, based on the distribution of training data. The distribution of reconstruction errors on the test dataset is shown in Figure 3. The vast majority of samples exhibit errors significantly below the threshold with most concentrated around 0.1, indicating that the Autoencoder successfully reconstructed normal samples. This shows the robustness of the model in accurately representing normal patterns while distinguishing potential anomalies.

**Figure 4:**
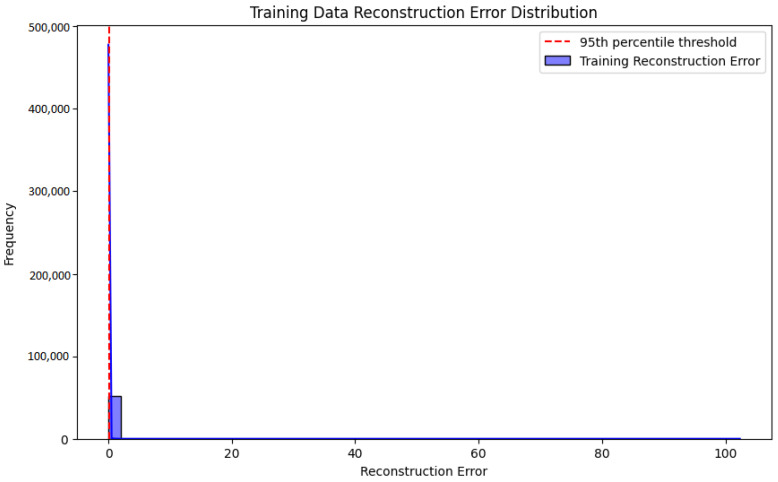
A histogram illustrating the distribution of reconstruction errors.

The histogram shown in Figure 4 illustrates the distribution of reconstruction errors for the training dataset with a red dashed line marking the threshold at the 95th percentile. The vast majority of the training samples exhibit low reconstruction errors, indicating that the Autoencoder has effectively learned the normal data patterns and reconstructs them with minimal discrepancies. The threshold at the 95th percentile is designed to capture outliers separating potential anomalies from the normal data distribution. Notably, there are no significant deviations beyond this threshold signifying that the model generalizes well to the training data and does not overfit. This distribution supports the robustness of the model in accurately identifying normal patterns while setting a clear criterion for anomaly detection.

### 4.2. LSTM Model

**Figure 5:**
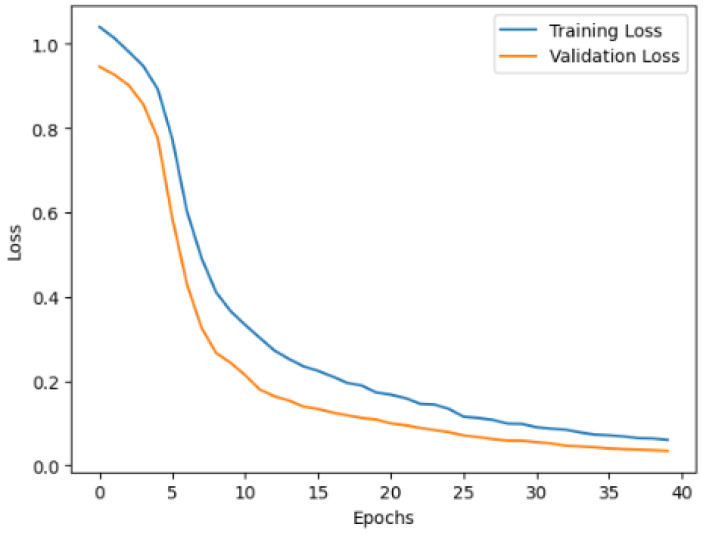
Training and validation loss of the LSTM model.

In the case of the LSTM-based Autoencoder, the training and validation loss steadily decreased, demonstrating effective learning of the underlying data patterns. Initially, the training loss was 95%, and the validation loss was 95%. By the final epoch, the training loss had reduced to 15.48%, and the validation loss reached a minimum of 6.56%, highlighting the capability of the model to reconstruct the input data effectively with minimal error. Figure 5 shows the performance of the model during training and validation phases. Initially, both losses are high and decrease as the model continues to learn. The gap between training and validation loss gradually narrows, indicating that the model generalizes well without overfitting. The smooth decrease in loss suggests stable convergence, highlighting that the model has learned the underlying patterns in the data effectively.

**Figure 6:**
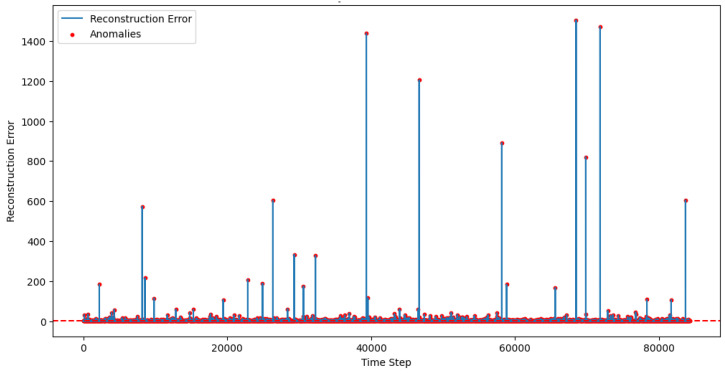
Anomaly detection in test data for LSTM-based Autoencoder.

Figure 6 shows anomaly detection in test data that illustrates the reconstruction error values over the time steps of a dataset. The blue line represents the reconstruction errors, with higher peaks indicating higher reconstruction discrepancies between input and reconstructed output. Red dots, which signify anomalies, highlight data points with significantly higher errors that surpass a set threshold. The largest spike in the plot is highly noticeable, signaling a clear anomaly in the dataset. This type of graph helps identify unusual or outlying behavior based on a predefined detection boundary, effectively separating anomalies from typical data patterns.

**Figure 7:**
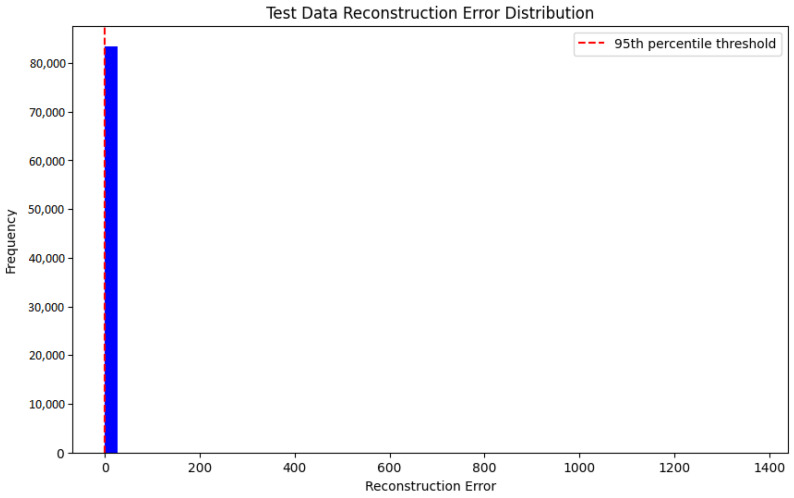
Distribution of reconstruction error on test data.

Figure 7 depicts the test data reconstruction error distribution. The histogram shows a concentration of errors tightly around low values, indicating that most data points exhibit relatively low reconstruction errors. The red dashed line indicates a threshold, commonly set at the 95th percentile, beyond which data points are deemed anomalous. This distribution helps verify that the model correctly differentiates normal data points from anomalies by establishing an appropriate threshold to identify deviations.

### 4.3. Convolutional Neural Network (CNN)

The results of training and validation of the CNN model over 40 epochs show a significant improvement in classification accuracy and consistent reduction in loss. During the first epoch, the model achieved a training accuracy of 96.04% with a loss of 0.04949, while the validation accuracy was 96.52% with a loss of 0.01132. These initial results indicate that the model started with relatively strong performance, likely due to effective initial weight initialization and thorough data preprocessing.

**Figure 8:**
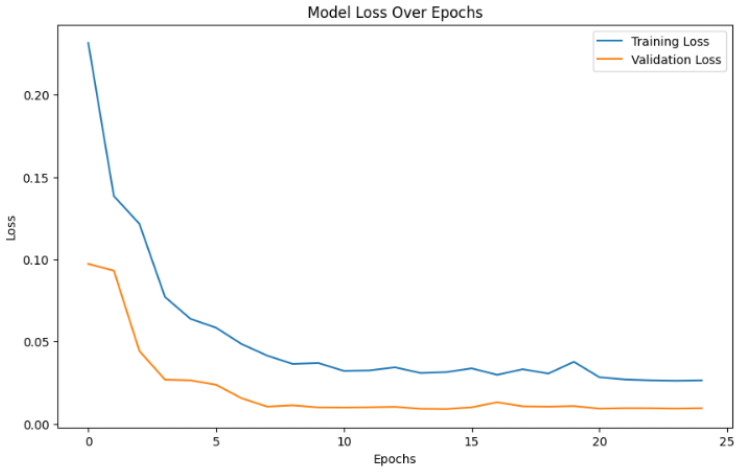
Performance of the CNN model during training and validation phases.

Figure 8 illustrates the training and validation loss of the model over 25 epochs. The training loss (blue line) decreases steadily indicating that the model is learning effectively from the training data. The validation loss (orange line) follows a similar downward trend, demonstrating good generalization to unseen data without signs of overfitting. Both curves converge toward low loss values, suggesting that the model achieves a strong performance on both the training and validation datasets by the final epoch.

As training progressed, the accuracy of the model steadily improved, reaching 94.39% in the second epoch and 97.12% by the fourth epoch, alongside reductions in training loss from 0.1646 to 0.0921. Similarly, the validation accuracy showed an upward trend, rising from 96.69% in the second epoch to an impressive 99.23% by the fourth epoch. This consistent improvement in validation performance suggests that the model was learning effectively without signs of overfitting, as indicated by the decreasing validation loss values, which dropped from 0.0996 to 0.0295.

**Figure 9:**
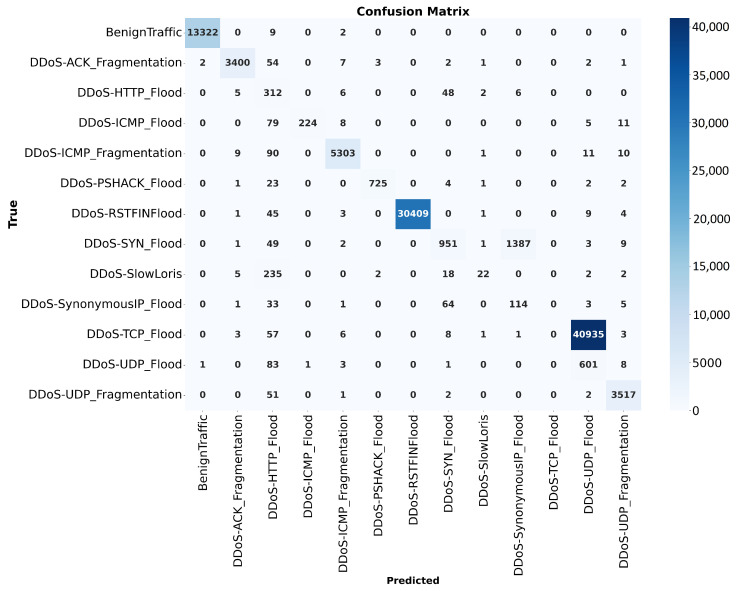
Confusion matrix of the CNN.

The classification report, as shown in Figure 9, summarizes the performance of the model across different traffic types in terms of precision, recall, and F1-score. High precision, recall, and F1-scores close to 1.00 are observed for most DDoS attack types, such as DDoS ICMPFlood and DDoS UDP Flood, as well as BenignTraffic, indicating strong predictive capability. However, the model struggles with specific attacks, like DDoS HTTP Flood and DDoS SlowLoris, achieving lower F1-scores of 0.42 and 0.14, respectively, due to reduced recall or precision. Despite this, the macro average F1-score of 0.88 suggests that the model performs well overall but requires improvement in detecting less frequent or more challenging attack types.

### 4.4. Hybrid Model

**Figure 10:**
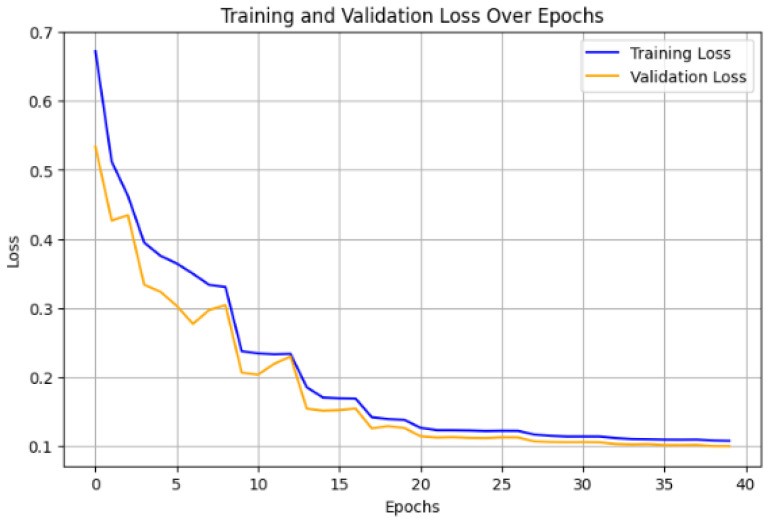
Performance of the hybrid model during training and validation phases.

The hybrid model that combines CNN, LSTM, and Autoencoder layers underwent a rigorous training process with 40 epochs for the feature extraction stage, followed by 40 epochs for the classification stage. The training began with a notable reduction in loss during the early epochs, indicating a strong ability to capture underlying patterns within the data. For example, during Epoch 1, the loss was relatively high at 0.1544 but quickly decreased, with a significant drop in validation loss to 0.0057, demonstrating early success in generalizing well on unseen data of the model. Over subsequent epochs, fluctuations in the loss function were observed, but the general trend indicated effective learning leading to substantial gains in accuracy. The graph shown in Figure 10 depicts the training and validation loss of the hybrid model over 40 epochs. Both training and validation loss exhibit a consistent decline, which indicates effective model learning and generalization. Initially, the training loss (blue line) decreases rapidly, which suggests that the model quickly captures significant patterns in the data. The validation loss (orange line) follows a similar trend, slightly lower than the training loss in later epochs, which indicates good generalization without overfitting. The losses stabilize around Epoch 40, converging to low values, demonstrating the model’s success in achieving robust performance on both the training and validation datasets.

The final stage involved classification, with accuracy improving progressively across 40 epochs. From an initial accuracy of 34.39% in Epoch 1, the model reached a test accuracy of 96.78% after 40 epochs. During this phase, the validation accuracy steadily increased, peaking at 96.60% by the last epoch. This steady improvement highlights the ability of the model to learn effectively across both training and validation datasets. The use of CNN layers likely aided in identifying spatial relationships, while LSTM layers contributed to learning temporal dependencies, thereby enhancing the overall prediction accuracy, as shown in Table 1.

**Figure 11:**
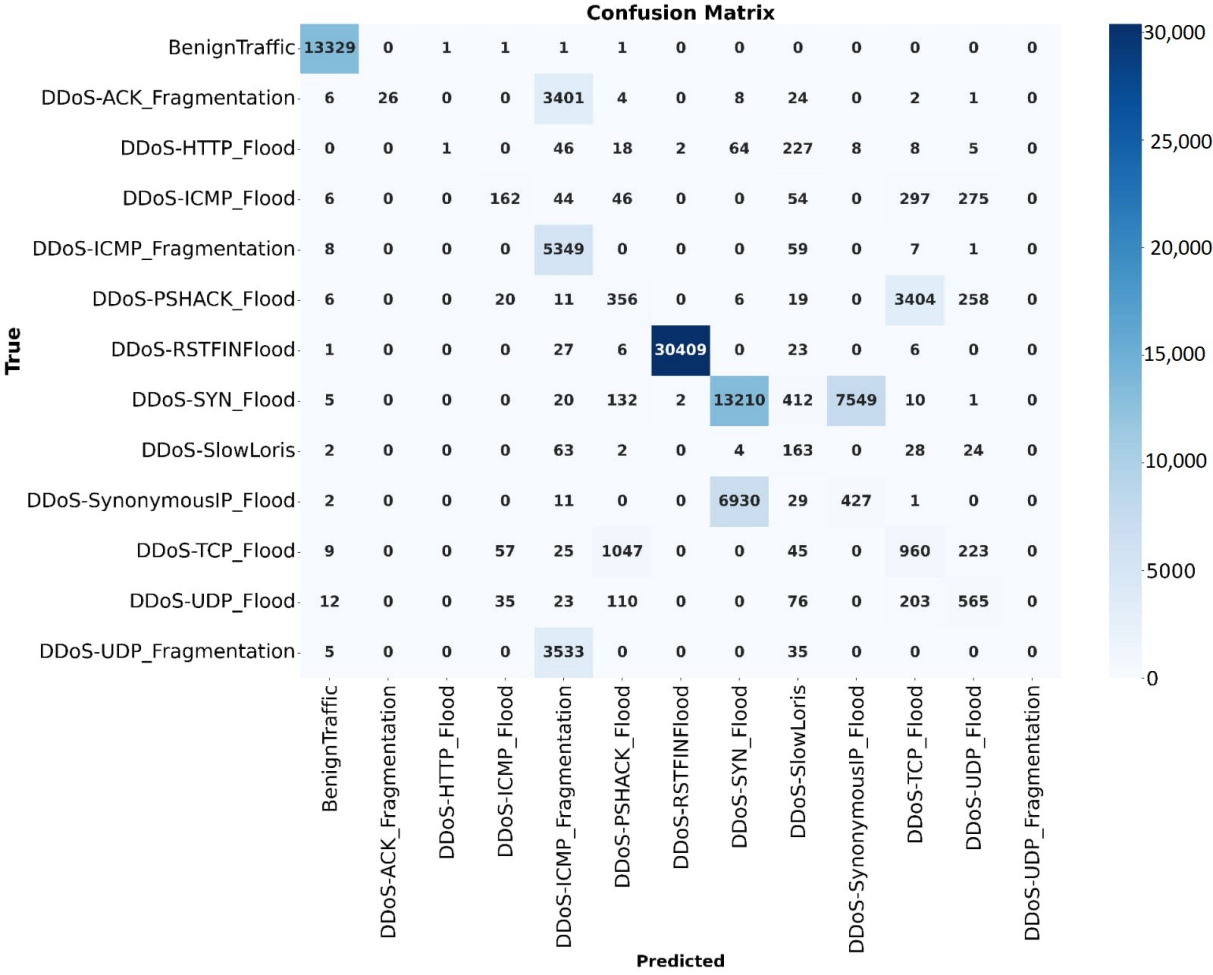
Confusion matrix of the hybrid model.

The classification report shown in Figure 11 provides further insights into the performance of the model on different classes. The average precision, recall and F1-score values, though somewhat lower due to poor detection in rare classes like ‘DDoS-UDP Fragmentation’, reflect the complexity of identifying a broad range of attack types. Conversely, classes such as ‘Benign Traffic’, ‘DDoS-ICMP Flood’, and ‘DDoS-UDP Flood’ were detected with near-perfect accuracy, achieving an F1-score of approximately 0.99. This indicates that while the hybrid model performs exceptionally well on dominant classes, there is still room for improvement in addressing imbalances and rare classes in the dataset.

The comparison of the accuracy of different models on the CICIOT2023 dataset is shown in Table 2. The results show the improvements achieved by the proposed architectures over the compared methods. Existing models, i.e., CNN, DNN, and LSTM [41], achieved accuracies of 90.64%, 89.88%, and 91.27%, respectively. CNN-LSTM, DNN and RNN models [42] achieved 87, 88, and 93% accuracies, respectively. The models (DNN, CNN, RNN) evaluated in [43] achieved 84.73, 94.30, and 95.89% accuracies, respectively. CNN, RNN, and LSTM models in [44] achieved accuracies of 92.21, 92.73, and 92.75%, respectively. The proposed hybrid model achieved 96.78% accuracy. The results show the effectiveness of the model in achieving higher accuracy compared to existing models.

### 4.5. Discussion

The presented results demonstrate the effectiveness of the proposed model in detecting and classifying DDoS attacks in IoT networks. This discussion covers the strengths, challenges, and potential areas of improvement of each model, along with an overall assessment of the proposed hybrid approach. The Latent Autoencoder model shows strong reconstruction capabilities, achieving better accuracy in anomaly detection. These results show the ability of the Autoencoder to effectively learn the patterns of normal IoT traffic. Using a 95th percentile threshold for the reconstruction error, the model was able to differentiate anomalous traffic accurately without significant overfitting. However, while the Autoencoder performs well on normal data, its dependence on the reconstruction threshold may limit its flexibility when encountering diverse attack types that do not fit predefined error boundaries. The LSTM Autoencoder, with sequential modeling capabilities, further improves anomaly detection by learning temporal dependencies in network traffic. Achieving an accuracy of 95%, this model demonstrated better precision in identifying irregularities in network behavior. The reconstruction error threshold proved effective for anomaly classification. However, occasional spikes in loss values suggest potential sensitivity to specific traffic patterns or noise within the data. Addressing this sensitivity through data augmentation or robust preprocessing techniques could further stabilize LSTM performance.

The CNN model shows improvements during the training and validation phases. Having better test accuracy and maintaining low validation loss, the CNN shows robustness in extracting spatial features from network traffic data. The ability to generalize well without overfitting shows the efficacy of convolutional layers in capturing spatial dependencies. The classification report shows better accuracy for dominant attack types, such as DDoS-ICMP Flood and DDoS-UDP Flood. However, challenges exist with rare attack classes, such as DDoS-HTTP Flood and DDoS-SlowLoris, where lower F1-scores were observed. This imbalance shows the need for additional measures, such as data rebalancing or oversampling of minority classes, to improve the detection accuracy across all categories.

The hybrid model, combining CNN, LSTM, and Autoencoder components, achieved an accuracy of 96.78%, showed its potential in integrating feature extraction, temporal dependency modeling, and dimensionality reduction. The hybrid approach captured complex relationships in IoT network traffic with robust learning capabilities. However, despite its overall balanced performance, the hybrid model faced challenges with rare attack types, suggesting a need for further refinement, potentially through ensemble techniques or additional layers focused on minority class detection.

## 5. Conclusions

This article presents and evaluates a hybrid model for detecting and classifying DDoS attacks in IoT networks. The results show the effectiveness of various deep learning approaches, including the Latent Autoencoder, LSTM Autoencoder, CNN, and a hybrid model combining these techniques. Each model was trained and evaluated based on the ability to detect anomalies, with performance metrics including accuracy, precision, recall, and F1-score. The hybrid model, integrating CNN, LSTM, and Autoencoder layers, showed improved results, achieving an accuracy of 96.78%. This performance shows the potential for complex attack detection in IoT networks, though further refinement is needed to improve detection for rare attack types. Overall, the results show the effectiveness of these models in real-world IoT environments, with the hybrid model showing the potential for future applications due to its ability to utilize both spatial and temporal dependencies. The success of these techniques shows their suitability in improving IoT network security against DDoS attacks. Future work will focus on addressing the limitations observed in the current models, particularly in the detection of less frequent DDoS attacks. Additionally, we will focus on training time and other issues related to class balancing or resampling strategies.

## Figures and Tables

**Table 1:** Summary of accuracy metrics for the hybrid model.

Metric	Value
Accuracy	96.78%
Loss	0.12
F1-Score	96%
Precision	96%
Recall	96%

**Table 2:** Comparison of different models’ accuracy.

Model	Accuracy (%)
CNN, DNN, LSTM [41]	90.64, 89.88, 91.27
CNN-LSTM, DNN [42]	87.0, 88.0, 93.0
DNN, CNN, RNN [43]	84.73, 94.30, 95.89,
CNN, RNN, LSTM [44]	92.21, 92.73, 92.75
Proposed Hybrid	96.78%

## Data Availability

The datasets used in this study are available publicly.
